# How to assess severe burnout? Cutoff points for the Burnout Assessment Tool (BAT) based on three European samples

**DOI:** 10.5271/sjweh.4093

**Published:** 2023-05-01

**Authors:** Wilmar B Schaufeli, Hans De Witte, Jari J Hakanen, Janne Kaltiainen, Robin Kok

**Affiliations:** 1 Department of Psychology, Utrecht University, The Netherlands.; 2 O2L, Research group Work, Organizational and Personnel Psychology (WOPP), KU Leuven, Belgium.; 3 Optentia Research Unit, North-West University, Vanderbijlpark, South Africa.; 4 Workability and Work Careers, Finnish Institute of Occupational Health, Helsinki, Finland.; 5 HumanTotalCare, Research & Business Development, Utrecht, The Netherlands.

**Keywords:** clinical burnout, ROC analysis, cross-national, burnout diagnosis

## Abstract

**Objective:**

Despite decades of burnout research, clinical validated cut-off scores that discriminate between those who suffer from burnout and those who don’t are still lacking. To establish such cut-off scores, the current study uses a newly developed questionnaire, the Burnout Assessment Tool (BAT) that consists of four subscales (exhaustion, mental distancing, and emotional and cognitive impairment). Separate cut-offs were computed for those at risk for burnout and those suffering from severe burnout for the original BAT-23 as well as for the shortened BAT-12.

**Methods:**

Relative operating characteristic (ROC) analyses were carried out using representative samples of healthy employees from The Netherlands (N=1370), Belgium (Flanders; N=1403) and Finland (N=1350). In addition, samples of employees who received a burnout diagnosis were used (N=335, 158 and 50, respectively).

**Results:**

The diagnostic accuracy of the BAT (area under the curve) ranges from good to excellent with the exception of mental distancing, which is fair. The country-specific cut-off values as well as their specificity and sensitivity are comparable to those of the pooled sample.

**Conclusions:**

In addition to country-specific cut-offs, general cut-offs can be used tentatively in other similar countries, pending future replication studies. Caution is warranted for using cut-offs for mental distance as the sensitivity and specificity of this subscale is relatively poor. It is concluded that the BAT can be used in organizational surveys for identifying employees at risk for burnout and, in clinical treatment settings, for identifying those with severe burnout, keeping in mind the tentativeness of the present cut-offs.

For decades, burnout has been a major issue in organizations and hence an important topic in occupational health. Two approaches exist that view burnout either as a multi-faceted crisis of employees with their work (1) or as a psychological disorder (2). This confusion about the nature of burnout is rooted in the history of the concept. It was introduced as a non-medical term for the crisis with work as experienced by human services professionals as a result of their emotionally demanding job (3). Traditionally burnout is measured with the Maslach Burnout Inventory (MBI), which includes three subscales that emerged from factor-analyses: emotional exhaustion, mental distancing (depersonalization), and reduced professional efficacy (4). In other words, the MBI resulted from an inductive, data-driven approach.

Because of its almost universal acceptance, the MBI soon became the gold standard, which implied that the concept of burnout became identical to the way it was measured. This circularity obstructed scientific progress because burnout could only be studied using the MBI, which suffers from several shortcomings. For instance, its practical use is hampered because the three subscale scores should not be combined into a composite score. This means that the MBI cannot be used for assessing burnout as an overall construct, which is important in an occupational health context. In some European countries, burnout is recognized as an occupational disease (5) which implies that it must be diagnosed as such so that employees are eligible for financial compensation and treatment.

Despite the fact that formally speaking the MBI cannot be used for assessing burnout as a psychological disorder, various attempts have been made to estimate the prevalence of severe burnout. The resulting confusion is illustrated by a systematic review of 182 studies, involving over 100 000 physicians from 45 countries, which found that 47 unique cut-off scores for MBI-burnout were employed (6). Overall burnout prevalence ranged from 0–80%, whereas emotional exhaustion, mental distancing, and low professional efficacy prevalence ranged from 0–86%, 0–90%, and 0–87%, respectively. In a similar vein, a more recent European systematic review of physician burnout including 56 studies from 41 countries found overall prevalence rates based on the MBI ranging from 2–72% (7). Needless to say, that these figures are uninformative and preclude definitive conclusions about the prevalence of burnout among – in this case – physicians. It is not surprising that both systematic reviews call for proper cut-off scores to assess burnout.

The current paper answers this call by establishing cut-off scores for a new burnout measure called the Burnout Assessment Tool (BAT) (8). Unlike the MBI, the BAT is founded on theoretical considerations, specifically the conceptual framework of burnout developed by Schaufeli & Taris (9). They followed in the footsteps of the great old man of psychological fatigue research, Edward Thorndike (10), who argued that the fundamental tenet of fatigue is “the intolerance of any effort” and theorized that burnout is a combination of the inability and unwillingness to no longer expend the necessary effort at work for proper task completion. In their view, “inability” manifests as a lack of energy, while “unwillingness” manifests as increased resistance, reduced commitment, a lack of interest, and disengagement. In fact, inability and unwillingness are two inseparable components of the burnout phenomenon, representing its energetic and motivational dimensions, respectively.

In addition to *exhaustion* (ie, severe loss of energy resulting in feelings of both mental and physical exhaustion) and *mental distancing* (ie, psychologically distancing oneself from work as indicated by a strong reluctance or aversion to work), the BAT includes two dimensions not included in the MBI: *cognitive impairment* (ie, memory problems, attention and concentration deficits, and poor cognitive performance) and *emotional impairment* (ie, intense emotional reactions and feeling overwhelmed by one’s emotions). The inclusion of these two dimensions reflects a growing understanding that cognitive and emotional impairment are components of burnout. Meanwhile, it has been established that burnout is also associated with decreased cognitive performance, which means that cognitive functions like attention, concentration, and working memory are impaired (11). Although burned-out employees’ functioning improves after two years, their cognitive performance remains inferior to that of healthy individuals (12). Furthermore, emotional regulation and burnout were found to be positively related (13), prompting Van Dam (2) to consider emotional impairment as a component of “clinical burnout.” Finally, unlike the MBI, professional efficacy was left out of the BAT because it appears to be the *result* of burnout rather than an essential component of it (9). A longitudinal study, for example, found that exhaustion and cynicism lead to a lack of professional efficacy (14).

In conclusion, the BAT is a theory-based, cutting-edge burnout measure that is based on reconceptualizing burnout as a work-related state of exhaustion that occurs among employees and is characterized by extreme tiredness, reduced ability to regulate cognitive and emotional processes, and mental distancing. In burnout, there is a lack of energy to properly regulate one’s emotions and cognitions; from this vantage point, emotional and cognitive impairment are distinct aspects of exhaustion. Mental distancing (ie, reducing motivation) is a coping mechanism for dealing with exhaustion, but it is deemed ineffective because it exacerbates rather than alleviates feelings of fatigue. In this sense, the energetic (exhaustion) and motivational (mental distancing) components of burnout are two sides of the same coin.

In contrast to the MBI, the BAT fulfills the measurement criteria according to the Rasch model and can therefore be considered a one-dimensional measure of burnout (15–17). This means that burnout, as assessed with the BAT, can be considered as syndrome; a set of four associated symptoms that occur together and refer to the same underlying condition. This applies likewise to the shortened, 12-item version of the BAT (18, 19). Moreover, measurement invariance of the BAT was demonstrated across seven countries, including the three countries of the current study (20). To be entirely sure, invariance analyses were carried out with only the three samples of the current study [The Netherlands, Belgium (Flanders region), and Finland], using the same analytical strategy. The results support measurement invariance across the three countries (detailed results are available on request by the first author)

To establish proper cut-off scores for the BAT, relative operating characteristic (ROC) analysis is employed (21). This allows to determine cut-off scores with optimal sensitivity and specificity when it comes to discriminating burnout-cases from non-cases (for more details see the Methods section). It is paramount for ROC analysis to include a group of employees who unequivocally suffer from burnout using an *independent, non-self-report* criterion such as an interview-based diagnosis by a trained professional.

Although some ROC studies with the MBI have been conducted, these were poorly designed. For instance, self-reports were used as basis for selecting a “burned-out” group thereby violating the assumption of independent assessment by another source (22–24). Only very few studies used a proper independent, non-self-report criterion for identifying burned-out employees. In one study, a consultant psychiatrist independently clinically assessed burnout among students according to the ICD-10 diagnostic criteria for work-related neurasthenia (25). The discriminatory power of the exhaustion, cynicism and professional efficacy subscales was high, with AUC-values of 0.90, 0.94, and 0.95, respectively. Also, the sensitivity and specificity of the cut-off scores was very good with values >0.90. In a similar vein, a study among Dutch employees who were treated for work-related psychological problems used the DSM-IV criteria for “undifferentiated somatoform disorder” supplemented with fatigue as the primary complaint to identify “clinical burnout” (26). A trained mental health professional carried out the assessment using a structured diagnostic DSM-interview. It appeared that only the AUC of the exhaustion subscale was more or less acceptable (0.64), whereas both other subscales showed poor AUC values (cynicism 0.55 and professional efficacy 0.51). Although the sensitivity of cut-off value for exhaustion is reasonable, its specificity is rather poor. Using a sample from an occupational health service, another Dutch study investigated the discriminatory power of MBI-burnout – the combination of exhaustion and cynicism – for future long-term sickness absence (27). This study concluded that MBI-burnout was not practically useful in identifying employees at high risk for long-term sickness (>41 days) in the next year. The AUC value of 0.60 for MBI-burnout was only slightly higher than chance (0.50).

In sum: using self-report criteria for identifying employees suffering from burnout recalls the fairy tale of Baron Von Münchhausen, who pulled himself out of the swamp by his own boots. The results of studies using this approach may seem impressive at first sight because of the high discriminatory power of the MBI, but this is clearly a wrong approach because it is tautological. Overall, the results of the few ROC studies that include proper groups of burned-out employees are mixed with most studies concluding that the MBI is not able to discriminate between those with and without burnout. So, it can be concluded that there is insufficient evidence for the utility of the MBI to assess severe burnout among employees.

The present study sought to establish cut-off scores for the BAT using ROC analyses that include samples suffering from severe burnout from three European countries: The Netherlands, Belgium (Flanders) and Finland. This offers the additional possibility to investigate the feasibility of similar cross-national cut-off values versus country-specific cut-offs.

## Method

### Samples and procedure

Healthy employee samples (N=1500) were randomly drawn from the Dutch and Belgian (Flemish) labor force in 2017 by a commercial surveying agency (*iVox*) in such way that they were representative of age, gender, and industry. In order to ensure that only healthy employees without burnout complaints were included, those who had been treated for burnout in the past five years were excluded (N=130 and N=97 for The Netherlands and Belgium, respectively). Thus leaving 1370 healthy Dutch [mean age 41.76, standard deviation (SD) 13.40, years; male=54%, female=46%] and 1403 healthy Belgian employees (mean age 41.28, SD 11.58, years; male=55%, female=45%) for the ROC analysis. In Finland, a randomized healthy population sample (N=1567) was collected in December 2019 and January 2020 by an established surveying agency (*Taloustutkimus Inc*). Those who had been treated for burnout in the past five years (N=163) or were not sure whether they had been treated (N=32) were excluded, resulting in 1372 healthy Finnish employees (mean age 46.09, SD 11.02, years; male=42.4%, female=57.6%). The healthy Dutch, Belgian and Finnish samples have been used before together with similar samples from Austria, Germany, Ireland and Japan in a previous cross-national study (13).

The BAT items were developed in Dutch and then translated into English with the assistance of a bilingual psychologist using a forward-backward translation procedure. A similar procedure was used for the Finnish BAT, which was translated and back translated from English with the help of a bilingual language consultant with work and organizational psychology experience.

Dutch employees with severe burnout were sampled from a nationwide occupational health service (ArboNed). All employees who listed sick due to psychological complaints between 1 March 2020 and 30 June 2021 completed an online questionnaire (N=5791) shortly after being absent due to illness. Within six weeks after they called in sick, but usually much earlier, employees were invited for a personal interview with an occupational physician. They were individually assessed according to the guidelines for work-related mental problems issued by the Royal Dutch Medical Association^1^ (28). In total, 335 employees received a burnout diagnosis from the occupational physician (mean age 40.79, SD 10.42, years; male=45%, female=55%).

Belgian employees with severe burnout were recruited from a study on the epigenetics of burnout and depression at KU Leuven (N=40) in 2018, supplemented the same year by a group from a Belgian burnout prevention and treatment center (N=27). Participants from both groups were included on the basis of a clinical interview in which similar diagnostic criteria as in the Dutch guidelines were used. In addition, 91 employees were included who completed an online survey – that was posted in 2018 on a website for burnout victims (www.Burn-Out.Vlaanderen.be) – and confirmed that they had recently received a burnout diagnosis by a medical or psychological professional. So overall, 158 Belgian employees suffering from severe burnout were included (mean age 42.28, SD 10.37, years; male=25%, female=75%).

The Finnish burnout sample (N=50) was based on employees who visited their occupational health company (*Mehiläinen*) between November 2020 and October 2021 because of severe burnout complaints and were selected after clinical interviews by an occupational psychologist, physician, or nurse. Similar diagnostic criteria were used as in the two other countries.

The Social and Societal Ethics Committee (SMEC) of KU Leuven approved this study on 16 June 2016 (reference number: G-2016 06 2027) as far as the Belgian and Dutch participants are concerned. For the Finnish participants, the Ethical Review Committee of the Finnish Institute of Occupational Health approved this study (record 7/2019). All methods were in accordance with the declaration of Helsinki and/or in accordance with relevant national/institutional guidelines.

### Measure

All participants filled in the 23-item version of the BAT that consists of four subscales: emotional exhaustion (8 items, eg, “*After a day at work, I find it hard to recover my energy*”); cognitive impairment (5 items, eg, “When I’m working, I have trouble concentrating”); emotional impairment (5 items, eg, “*At work I may overreact unintentionally*”); and mental distancing (5 items, eg, “*I feel a strong aversion towards my job*”). The short version of the BAT includes 12 items, 3 for each subscale. Items are scored on a 5-point rating scale ranging from never (1) to always (5).

### Analysis

Using ROC analysis, an optimum cut-off value can be calculated to discriminate burnout cases from non-cases, considering both specificity (the probability of a negative result, meaning that someone is correctly *not* identified as a burnout case – *true negative rate*) and sensitivity (the probability of a positive result, meaning that someone is correctly identified as a burnout case *– true positive rate*). ROC curves were computed for different cut-off values of the BAT-23 and the shortened BAT-12 by using SPSS version 27, (IBM Corp, Armonk, NY, USA). Cut-offs for subscales were only computed for the BAT-23 and not BAT-12. For individual assessment, internal consistency coefficients (Cronbach’s α) should be ≥0.90 (29), which is the case for the subscales of the BAT-23 but not the BAT-12 (30). We calculated two different kinds of cut-off values that allow to distinguish between three groups, using the so-called traffic light model (31): (i) a green, non-burnout group, (ii) an orange group at risk for burnout and (iii) a red, burned-out group.

The diagnostic accuracy of the BAT was assessed with the AUC, whereby a value of 0.50 represents chance level, and 0.90 is considered “excellent”, 0.80 “good”, 0.70 “fair”, and 0.60 “poor” (32). An AUC of 0.80 means that a randomly chosen pair of participants (one with and one without burnout) would be classified correctly 80% of the time. No critical values for specificity and sensitivity exist because these depend on the use of the questionnaire under study. In our case we used a minimum specificity of 0.90 for identifying the red, burnout group. This implies that the likelihood for obtaining a false positive result is <10%, which is considered to be acceptable for clinical use of the BAT in a treatment setting. After all, when selecting employees for burnout treatment, the likelihood for selecting employees who are *not* burned-out (false positives) should be minimized.

A BAT score is considered at risk (orange) when the cut-off corresponds with the score at which the sum of specificity and sensitivity is largest (33). This is indicated by the *Youden index* and reflects the maximum vertical distance of the ROC curve from the diagonal line. A cut-off that is based on the Youden index is considered to be adequate for screening purposes in organizations, where in addition to specificity also sensitivity matters because *all* possible burnout cases should be included. For an orange cut-off, a value of ≥0.70 is considered to be sufficient.

## Results

Table 1 shows the means, SD, and internal consistencies of the BAT in the three national samples of healthy and burned-out employees. As to be expected, the burnout sample scores higher on *all* BAT (sub)scales than the healthy sample (all P<0.001; except mental distance in the Dutch sample, P<0.01). Furthermore, the internal consistency of the BAT is good with virtually all values of α>0.80, and with the remaining α≥0.75. Half of the α in table 1 are equal to or greater than 0.90.

**Table 1:** Burnout Assessment Tool (BAT) means, standard deviations (SD) and internal consistencies across countries and healthy and burnout samples [SD=standard deviation; α=Cronbach alpha].

			Netherlands			Belgium			Finland			Pooled	
			Healthy (N=1370)	Burnout (N=334)		Healthy (N=1403)	Burnout (N=158)		Healthy (N=1372)	Burnout (N=50)		Healthy (N=4145)	Burnout (N=542)
BAT-23													
	Exhaustion												
		Mean	2.32	3.64		2.23	3.78		2.42	3.77		2.32	3.82
		SD	0.85	0.73		0.74	0.89		0.69	0.41		0.77	0.67
		α	0.84	0.90		0.92	0.85		0.89	0.75		0.92	0.88
	Mental distance												
		Mean	2.11	2.28		1.95	3.07		1.98	3.88		2.01	2.86
		SD	0.93	0.85		0.38	0.86		0.79	0.79		0.86	0.89
		α	0.92	0.79		0.91	0.85		0.89	0.88		0.91	0.81
	Cognitive impairment												
		Mean	2.07	3.41		2.10	3.35		2.06	3.37		2.08	3.39
		SD	0.84	0.80		0.68	0.69		0.67	0.53		0.74	0.75
		α	0.94	0.91		0.92	0.89		0.90	0.78		0.92	0.90
	Emotional impairment												
		Mean	1.96	3.07		1.78	3.02		1.82	3.11		1.86	3.06
		SD	0.90	0.87		0.68	0.81		0.61	0.68		0.74	0.83
		α	0.94	0.89		0.89	0.88		0.85	0.84		0.90	0.88
	Total												
		Mean	2.14	3.36		2.04	3.37		2.12	3.39		2.10	3.35
		SD	0.79	0.65		0.62	0.53		0.58	0.43		0.67	0.60
		α	0.97	0.93		0.95	0.90		0.94	0.88		0.96	0.92
BAT-12 total													
	Mean		2.10	3.26		1.99	3.30		2.06	3.30		2.05	3.28
	SD		0.80	0.66		0.64	0.57		0.57	0.46		0.68	0.61
	α		0.95	0.87		0.92	0.84		0.89	0.81		0.92	0.87

Table 2 displays the diagnostic accuracy (AUC) for the BAT across three national samples, as well as the combined, pooled sample.

**Table 2:** Area under the curve (AUC) [BAT=Burnout Assessment Tool; CI=confidence interval].

		Netherlands			Flanders			Finland			Total sample	
		AUC	95% CI		AUC	95% CI		AUC	95% CI		AUC	95% CI
BAT-23												
	EX	0.91	0.89–0.93		0.94	0.92–0.95		0.95	0.93–0.96		0.92	0.91–0.93
	MD	0.69	0.66–0.72		0.83	0.79–0.86		0.84	0.80–0.89		0.76	0.74–.078
	CI	0.87	0.85–0.89		0.90	0.87–0.92		0.94	0.91–0.96		0.89	0.88–0.90
	EI	0.81	0.78–0.83		0.88	0.85–0.91		0.91	0.87–0.96		0.85	0.84–.087
	Total	0.87	0.85–0.89		0.94	0.92–0.95		0.96	0.94–0.97		0.91	0.90–.092
BAT-12												
	Total	0.87	0.85–0.89		0.93	0.91–.95		0.96	0.94–.097		0.91	0.89-0.92

**Figure 1:**
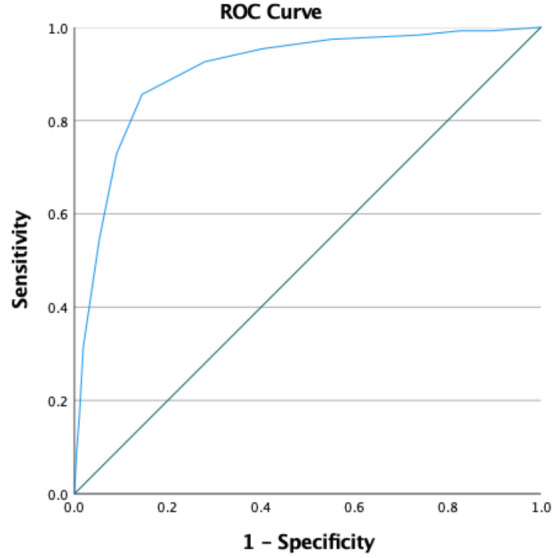
ROC-curve for the total-score of the BAT-23 in the pooled sample. Diagonal segments are produced by ties.

As can be seen from table 2, the diagnostic accuracy of the BAT-23 and its subscales as well as the BAT-12 is either good (≥0.80) or excellent (≥0.90). The only exception is mental distance with a fair accuracy in the total sample (≥0.70) and approaching this criterion in the Dutch sample (0.69). Virtually all lower bounds of the 95% CI are >0.80 whereas only for mental distance in The Netherlands, this lower bound is <0.70. By way of illustration, figure 1 shows the AUC for the total BAT-23 score in the pooled sample.

Table 3 displays the orange (at risk) and red (burnout) BAT cut-off scores across three national samples and the pooled sample.

**Table 3:** Cut-off values for the Burnout Assessment Tool (BAT) and its subscales [EX=exhaustion; MD=mental distance; CI=cognitive impairment; EI=emotional impairment]. *Note*: A table of all operating characteristics (sensitivity, specificity, 1-specificity and Youden-index for each BAT score) is available upon request from the first author.

		Cut-off	Netherlands	Belgium	Finland	Pooled	Max ∆
BAT-23							
	EX	Orange	3.06	3.06	3.06	3.06	0.00
		Red	3.44	3.31	3.31	3.31	0.13
	MD	Orange	2.10	2.30	2.30	2.10	0.00
		Red	3.50	3.10	3.10	3.30	0.20
	CI	Orange	2.70	2.70	2.70	2.70	0.00
		Red	3.10	3.10	3.10	3.10	0.00
	EI	Orange	2.10	2.10	2.30	2.30	0.20
		Red	3.10	2.90	2.70	2.90	0.20
	Total	Orange	2.59	2.59	2.67	2.59	0.08
		Red	3.20	3.02	2.89	3.02	0.18
BAT-12 total		Orange	2.54	2.54	2.63	2.54	0.09
		Red	3.13	2.96	2.79	2.96	0.17

The right-most column of table 3 shows the maximum difference between any of the national cut-offs and the pooled sample. In four cases, cut-offs are identical across countries, whereas in all remaining cases the maximum difference is ≤0.20. Practically speaking, considering that a 5-point scale was used, these differences are considered to be small, so that instead of using nation-specific cut-offs, values that are based on the pooled sample can be used just as well as cut-offs.

Tables 4 and 5 display the sensitivity and the specificity of the cut-offs using country-specific and overall values that are based on the pooled sample, respectively.

**Table 4:** Sensitivity (SENS) and specificity (SPEC) using country-specific values for the Burnout Assessment Tool (BAT) [EX=exhaustion; MD= mental distance; CI=cognitive impairment; EI=emotional impairment]. *Note*: A table of all operating characteristics (sensitivity, specificity, 1-specificity and Youden-index for each BAT score) is available upon request from the first author. **Bold denotes <0.70.**

		Cut-off	Netherlands		Belgium		Finland	
			SENS	SPEC	SENS	SPEC	SENS	SPEC
BAT-23								
	EX	Orange	0.89	0.82	0.90	0.88	0.96	0.84
		Red	0.76	0.90	0.81	0.92	0.84	0.90
	MD	Orange	0.76	**0.56**	0.81	0.71	0.88	0.71
		Red	**0.22**	0.92	**0.49**	0.90	**0.46**	0.91
	CI	Orange	0.82	0.76	0.83	0.82	0.90	0.84
		Red	**0.65**	0.90	0.88	0.94	0.76	0.94
	EI	Orange	0.87	**0.64**	0.89	0.72	0.92	0.80
		Red	**0.49**	0.90	**0.60**	0.92	0.76	0.92
	Total	Orange	0.90	0.72	0.94	0.83	0.96	0.87
		Red	**0.61**	0.90	0.73	0.92	0.90	0.91
BAT-12 total		Orange	0.88	0.72	0.90	0.83	0.98	0.82
		Red	**0.60**	0.90	0.72	0.90	0.72	0.94

**Table 5:** Sensitivity (SENS) and specificity (SPEC) using pooled values for the Burnout Assessment Tool (BAT). *Note*: A table of all operating characteristics (sensitivity, specificity, 1-specificity and Youden-index for each BAT score) is available upon request from the first author. **Bold denotes <0.70.**

		Cut-off	Netherlands			Belgium			Finland			Pooled	
			SENS	SPEC		SENS	SPEC		SENS	SPEC		SENS	SPEC
BAT-23													
	Exhaustion	Orange	0.89	0.82		0.90	0.88		0.96	0.84		0.90	0.85
		Red	0.80	0.87		0.81	0.92		0.84	0.90		0.80	0.90
	Mental distance	Orange	0.76	**0.56**		0.86	**0.64**		0.92	**0.63**		0.80	**0.61**
		Red	**0.25**	0.89		**0.40**	0.93		**0.46**	0.91		**0.30**	0.92
	Cognitive impairment	Orange	0.82	0.76		0.83	0.82		0.90	0.84		0.83	0.81
		Red	*0.65*	0.90		0.88	0.94		0.76	0.94		0.76	0.92
	Emotional impairment	Orange	0.81	**0.68**		0.79	0.81		0.92	0.80		0.81	0.76
		Red	**0.60**	0.80		**0.60**	0.92		**0.68**	0.95		**0.61**	0.89
	Total	Orange	0.90	0.72		0.94	0.83		0.98	0.81		0.92	0.79
		Red	0.73	0.87		0.73	0.92		0.84	0.93		0.74	0.91
BAT-12 total		Orange	0.88	0.72		0.90	0.83		0.98	0.82		0.89	0.79
		Red	0.71	0.82		0.72	0.90		0.72	0.94		0.71	0.89

As can be seen from table 4, most values for specificity and sensitivity are satisfactory (≥0.70). However, mental distance performs rather poorly, particularly as far as the sensitivity of the red cut-off is concerned, which in all three national samples is lower than chance (ie, 0.50). Moreover, in the Dutch sample the sensitivity of all red BAT cut-offs – except for exhaustion – is rather poor, and so is the red cut-off for emotional impairment in the Belgian sample. In contrast to some subscales of the BAT-23 (notably mental distance and, to a somewhat lesser degree, emotional impairment), the specificity and sensitivity of the total BAT-23 and BAT-12 is sufficient (except the sensitivity of the red cut-off in The Netherlands). Overall, 86% of the values in table 4 are satisfactory. Interestingly the sensitivity and specificity of the long and short versions of the BAT are quite similar.

As displayed in table 5, most values for specificity and sensitivity that are based on the pooled sample are satisfactory (≥0.70), except for the specificity of the orange and the sensitivity of the red cut-offs of mental distance in all samples, the specificity of the red cut-offs for emotional impairment in all samples, and the sensitivity of the red cut-off of cognitive impairment in The Netherlands. Like in table 4, the sensitivity and specificity of the total BAT-23 and BAT-12 are satisfactory, now also with values ≥0.70 in the Dutch sample. Overall, 85% of the values in table 5 are satisfactory.

Taken together, it seems that the pattern is similar in both tables. This means that applying pooled, general cut-off values in each country yields comparable results in terms of sensitivity and specificity (table 5) as applying country-specific cut-offs (table 4). In the discussion we elaborate on this result.

## Discussion

The current study sets out to establish clinically validated cut-off scores for the long (8) and short (18) versions of the BAT. This questionnaire conceives burnout as a *syndrome*, meaning that its four components (exhaustion, mental distance, and cognitive and emotional impairment) are interrelated and refer to a common underlying condition (15). This implies that in addition to subscale scores also a composite, overall burnout score can be used (8, 16–19). We analyzed samples from three different European countries: The Netherlands, Belgium (Flanders) and Finland. Also, the pooled sample was analyzed since it was demonstrated that BAT is invariant across these three countries, so that pooling is justified (20).

Our study boasts three main results. First, ROC-analyses show that the diagnostic accuracy (AUC) of the BAT and its four components is either good or excellent, except for mental distancing in the Dutch sample, which is fair. In fact, for each pair of employees, the BAT correctly identifies the one with burnout in about 80% (lower bound) to 90% (upper bound) of the cases (for mental distance this is about 70–80%). Second, the cut-off values for those at risk and those experiencing severe burnout differ only slightly across the three countries. That is, instead of nation-specific cut-offs, the same cut-offs based on the pooled sample could be used across the three countries. Taking this a step further, it implies that these general BAT cut-offs may be applied in other countries as well. Of course, this needs to be confirmed, but for the time being, our pooled BAT cut-offs can be used provisionally in other European countries similar to those in the current study.

Third, general cut-offs perform similarly well in terms of sensitivity and specificity compared to country-specific cut-offs, except for mental distancing, so that cut-offs for this subscale should be used with caution. Although the sensitivity of the red cut-off of emotional impairment (range 0.60–0.68) does not satisfy our criterion (≥0.70), it is well above chance level (0.50). Quite importantly, the sensitivity and specificity of the total BAT is good and rather similar for both versions (23 versus 12 items). This means that the short version of the BAT can be used without loss of sensitivity or specificity.

It appears that the mental distancing subscale performs rather poorly in discriminating burnout cases from non-burnout cases. The reason might be that burnout is first and foremost characterized by exhaustion. This is illustrated by a recent consensus study among 50 experts from 29 countries who came up with a rather restricted definition of burnout, essentially limiting it to mere exhaustion (34). Cognitive and emotional impairment can be seen as specific manifestations of exhaustion, namely as fatigue induced, diminished functional capacity to adequately regulate cognitive and emotional processes, respectively. Seen from this perspective, mental distance is not part of an exhaustion-based definition of burnout. Another reason that might particularly apply to the Dutch sample in which mental distancing performs poorest, is the nature of the burnout sample. As can be seen from table 1, the level of mental distancing is much lower in the Dutch burnout sample compared to other countries, and also compared to the other subscales of the BAT. Please note that the Dutch burnout sample consists of employees who have called in sick and were subsequently diagnosed by an occupational physician (a mandatory procedure), whereas in the other two samples employees took the initiative to approach a professional (a voluntary procedure). Obviously mental distancing plays a less prominent role in calling in sick voluntarily.

### Strengths and weaknesses

The current study includes samples from three European countries. On the positive side, the healthy samples were relatively large and representative for the country’s workforce. However, the size and the procedure followed for the burnout samples differed between countries. The Dutch sample included employees who had called in sick for mental reasons and were subsequently interviewed by an occupational physician. The Belgian sample consisted of employees who either received a burnout diagnosis from a professional as part of an ongoing research project or indicated that they recently received a burnout diagnosis from a health professional. The Finnish sample comprised employees who had visited their occupational health services because of burnout complaints and were asked to fill the online BAT survey. Hence, we cannot be completely sure that we sampled identical burnout cases across countries, so caution is warranted by comparing the three countries. Although it is assumed that similar diagnostic guidelines were used for assessing burnout in all three countries, we were not able to check this. For legal and practical reasons, it was not possible to evaluate the inter-rater reliability of the burnout assessments. So, future research is needed to establish the reliability of the diagnostic burnout interview.

We recommend that in addition – or instead of – country-specific cut-offs, general cut-offs can be used that are based on a pooled multiple-country sample. Strictly speaking this applies only to the three countries involved in the current study. Yet, these general cut-offs might be used – at least for the time being – in other countries as well, particularly where the BAT has been successfully validated such as in Brazil and Portugal (17), Romania (19), Germany, Austria, Ireland and Japan (20), Italy (35), South Africa (36), Ecuador (37), Turkey (38). However, future studies in these – and other countries – should corroborate our general cut-off values.

Finally, we would like to stress that the cut-off scores for the BAT, as reported in this paper, are preliminary and should therefore be used tentatively. Future well-designed studies that follow the guidelines for diagnostic accuracy studies (39) are highly recommended to validate the cut-offs for the BAT that are proposed in the current study.

### Practical implications

Since we found its diagnostic accuracy to be very good, the BAT can be basically used for discriminating between employees with and without burnout. We propose using the BAT-23 in a clinical setting, particularly for identifying those with severe symptoms who suffer from burnout disorder (2). They may benefit from specific treatment, such as cognitive behavioral therapy (40). Using the traffic light model (31), individuals can be classified as; (i) not-suffering from burnout (*green*); (ii) at-risk for burnout (*orange*); and (iii) suffering from severe burnout (*red*). The red cut-off is recommended as a criterion for burnout treatment because of its high specificity of around 90%. This means that the likelihood of false positives (those who are identified as burnout but do *not* suffer from it) is relatively low (about 10%). This is particularly relevant when resources for treatment are scarce. In addition to the overall BAT score the subscales, can be used for a more nuanced picture. It should be noted, however, that for mental distance the sensitivity of the red cut-off is poor, so that this subscale is not able to detect those who actually distance themselves mentally from their work. So many “real” cases are missed, although those cases who *are* identified suffer from severe distancing. For that reason, cut-offs for mental distancing should be used with caution. Needless to say, that for a comprehensive assessment of burnout additional information is needed from an anamnestic interview. Nevertheless, the BAT may be useful for estimating the seriousness of burnout complaints in occupational health settings. Needless to say, the BAT should be used in accordance with the current ethical principles and guidelines for psychological test use.

Furthermore, it is recommended to use to BAT-12 as a screening device in organizations for identifying employees who are at-risk for burnout, so that they can benefit from preventive measures. Individual BAT scores are private and should only be disclosed to screening participants. Only aggregated information about BAT scores should be reported to the organization, ensuring a balance of organizational awareness of burn-out symptoms and participant anonymity. For screening, either the orange or the red cut-off can be used depending on whether one wants to cast the net wider or not. In the former case, using the orange cut-off, the likelihood of selecting those at-risk is high (sensitivity about 0.90), whereas the likelihood of *not* selecting those who should have been selected (false negatives) is low (about 10%). In the latter case of casting a tighter net by using the red cut-off, the likelihood of false positives is lower, whereas that of false negatives is higher. This makes particularly sense when the resources for preventive measures are scarcer.

### Concluding remarks

We realize that there is an ongoing debate as to whether or not burnout is a mental disorder (41). To date, most emphasis has been placed on relatively mild symptoms of employees who are still working, while those with severe symptoms have been overlooked. Irrespective of the outcome of the debate, more knowledge about severe burnout is needed and the BAT might be useful to identify this group in future research. In addition, in some countries burnout is officially recognized as an occupational disease which calls for treatment and legally obliges organizations to implement preventive measures, including screening. The proposed cut-offs of the BAT – albeit used with caution – may assist health professionals and organizations to take informed decisions to identify employees most eligible for treatment or preventions programs.
